# Perioperative Management of Immune Thrombocytopenic Purpura in Cardiac Surgery: A Systematic Review of the Literature in Urgent and Elective Settings

**DOI:** 10.7759/cureus.103039

**Published:** 2026-02-05

**Authors:** K Said, Hicham Kbiri, Hakim El Baraka, Noureddine El Fassiki, Ayoub Bouchama, Chakib Chouikh, Naoufal Elghoul, Bassam Bencharfa, Jihane Bouzari, Mohammed Fajri, Hicham Sallahi, Omar Margad, Adbelatif Benbouha, Redouane Roukhsi, Hatim A El Ghadbane, Aznag Mohamed Amine, Mehdi Nabil, Issam Serghini, Hamza Najout, Monsif Salek, Abdellatif Chlouchi, Amine Bentaher, Ramiz Ballouk, Mourad Ababou, Hatim Belfquih, Najib Bouhabba, Monsef Elabdi, Abdelmajid Bouzerda, Ali Khatouri, Youssef Qamouss, Mohamed Zyani

**Affiliations:** 1 Anesthesiology and Reanimation, Mohammed V Military Training Hospital, Rabat, MAR; 2 Anesthesiology, ICU, and Emergency, Avicenna Military Hospital, Marrakesh, MAR; 3 Anesthesiology and Reanimation, Avicenna Military Hospital, Marrakesh, MAR; 4 Anaesthesiology, Avicenna Military Hospital, Marrakesh, MAR; 5 Anesthesiology and Critical Care, Mohammed V Military Training Hospital, Rabat, MAR; 6 Department of Orthopedic Surgery and Traumatology, Faculty of Medicine and Pharmacy, Mohammed V Military Teaching Hospital (HMIMV) - Mohammed V University, Rabat, MAR; 7 Polyvalent Intensive Care Unit (ICU), Marrakesh International Clinic, Marrakesh, MAR; 8 Anesthesiology, Marrakesh International Clinic, Marrakesh, MAR; 9 Orthopaedics and Traumatology, Avicenna Military Hospital, Marrakesh, MAR; 10 Trauma and Orthopedics, Faculty of Medicine, Cadi Ayyad University, Marrakesh, MAR; 11 Trauma and Orthopedics, Faculty of Medicine and Pharmacy of Casablanca, Hassan II University of Casablanca, Casablanca, MAR; 12 Radiology, Avicenna Military Hospital, Marrakesh, MAR; 13 Cardiovascular Anesthesioloy and ICU, Mommed V Training Military Hospital, Rabat, MAR; 14 Clinical Hematology, Avicenna Military Hospital, Marrakesh, MAR; 15 Anesthesiology and Reanimation, Military Hospital of Avicenne, Marrakesh, MAR; 16 Department of Anesthesia Anesthesia Intensive Care Unit (ICU), Mohammed V Military Teaching Hospital (HMIMV) - Mohammed V University, Rabat, MAR; 17 Faculty of Medicine, Pharmacy and Dentistry, Sidi Mohamed Ben Abdellah University, Fes, MAR; 18 Diagnostic and Interventional Radiology Department, Moulay Ismail Military Hospital, Meknes, MAR; 19 Anesthesiology ICU, Congenital Cardiovascular Surgery, Louis Pradel Hospital – part of the Hospices Civils de Lyon (HCL), Lyon, MAR; 20 Anesthesiology, Mohammed V Military Training Hospital, Rabat, MAR; 21 Radiology, Moulay Ismail Military Hospital, Meknes, MAR; 22 Interventional Cardiology, Hassan II Military Hospital, Laayoune, MAR; 23 Cardiothoracic ICU, Marie-Lannelongue Hospital, Paris, FRA; 24 Department of Neurosurgery, Avicenne Military Hospital, Cady Ayyad University, Marrakesh, MAR; 25 Department of Neurosurgery, Mohammed V University, Rabat, MAR; 26 Anesthesiology and Perioperative Medicine, Military Hospital Oued Eddahab, Agadir, MAR; 27 Trauma and Orthopedics, Hassan II Military Hospital, Laayoune, MAR; 28 Cardiology, Avicenna Military Hospital, Marrakesh, MAR; 29 Cardiology, Avicenna Training Military Hospital, Marrakesh, MAR; 30 Anesthesia and Critical Care, Avicenna Military Hospital, Marrakesh, MAR; 31 Internal Medicine, Avicenna Military Hospital, Marrakesh, MAR; 32 Internal Medicine, Faculty of Medicine and Pharmacy, Marrakesh, Marrakesh, MAR

**Keywords:** cardiac surgery, cardiopulmonary bypass, immune thrombocytopenia, immune thrombocytopenic purpura, intravenous immunoglobulin, patient blood management, platelet transfusion, thrombopoietin receptor agonist, tranexamic acid

## Abstract

Perioperative management of immune thrombocytopenic purpura (ITP) in cardiac surgery represents a rare yet formidable clinical challenge driven by immune-mediated platelet dysfunction, obligatory systemic anticoagulation, and the profound hemostatic derangements induced by cardiopulmonary bypass. We systematically synthesized the available evidence on perioperative strategies and outcomes in adults with ITP undergoing cardiac surgery in elective and urgent or emergent settings by reviewing PubMed/MEDLINE, Embase, Scopus, Web of Science, Cochrane CENTRAL, ClinicalTrials.gov, and the WHO International Clinical Trials Registry Platform from inception through August 26, 2025. Nineteen studies comprising approximately 74 patients, predominantly with severe thrombocytopenia, were identified, including three observational cohorts and sixteen case reports or series. Reported management approaches centered on rapid immunomodulation with intravenous immunoglobulin and/or corticosteroids, selective adjunctive platelet transfusion, antifibrinolytic therapy, thrombopoietin receptor agonists in selected cases, and carefully monitored cardiopulmonary bypass anticoagulation, most commonly using unfractionated heparin with activated clotting time guidance; bivalirudin was described in isolated reports. Across studies, perioperative bleeding occurred in approximately 32% of patients, platelet transfusion in 45%, reoperation for bleeding in 8%, thrombotic complications in 6%, and perioperative mortality in 7%, with consistently inferior outcomes observed in urgent and emergent procedures. Although the evidence base remains largely case-derived and heterogeneous, these data support a coherent multimodal perioperative strategy integrating rapid platelet-directed immunotherapy, patient blood management-guided hemostasis, antifibrinolytic use, and vigilant anticoagulation, while underscoring the urgent need for prospective, multicenter registries with harmonized outcome definitions to inform standardized, evidence-based perioperative pathways for this high-risk population.

## Introduction and background

Immune thrombocytopenic purpura (ITP) is an acquired autoimmune disorder characterized by isolated thrombocytopenia (platelet count <100 × 10⁹/L) caused by immune-mediated platelet destruction and impaired platelet production. Autoantibodies targeting platelet glycoproteins, Fc receptor-mediated reticuloendothelial clearance (predominantly splenic), and T-cell-mediated cytotoxicity drive heterogeneous bleeding phenotypes and variable clinical severity [1-3]. While many patients exhibit only mild mucocutaneous bleeding, exposure to major surgery and systemic anticoagulation may precipitate clinically consequential hemorrhage. ITP is uncommon among candidates for cardiac surgery, with its prevalence inferred largely from isolated case reports and small series rather than population-level surgical registries.

Cardiac surgery, particularly when cardiopulmonary bypass (CPB) is required, constitutes one of the most hemostatically challenging operative environments. CPB promotes platelet activation, consumption, and qualitative dysfunction; postoperative thrombocytopenia after CPB is independently associated with increased morbidity and mortality [4]. Mandatory systemic anticoagulation (typically unfractionated heparin) further narrows the safety margin between bleeding and thrombosis, while contemporary care increasingly follows patient blood management (PBM) principles to minimize allogeneic exposure and target hemostatic interventions [5-7].

In ITP, perioperative decision-making is uniquely complex: transfused platelets may be rapidly cleared, limiting durability, whereas platelet-augmenting therapies (intravenous immunoglobulin (IVIG), corticosteroids, and thrombopoietin receptor agonists (TPO-RAs)) may transiently increase thrombotic risk, an especially acute trade-off when surgery is urgent and optimization time is limited [1-3]. Consequently, practice has largely been informed by case-based literature spanning coronary artery bypass grafting (CABG), valve surgery, and complex aortic procedures [8-12]. We therefore conducted a systematic review to synthesize perioperative strategies and associated outcomes for adults with ITP undergoing cardiac surgery in elective versus urgent/emergent settings.

## Review

Methods

This systematic review was conducted and reported in accordance with the Preferred Reporting Items for Systematic reviews and Meta-Analyses (PRISMA) 2020 statement.

Eligibility Criteria (PICOS)

Population: Adults (≥18 years) with ITP undergoing cardiac surgery (elective, urgent, or emergent)

Interventions: Strategies addressing thrombocytopenia and hemostasis (platelet transfusion, IVIG, corticosteroids, TPO-RAs), antifibrinolytics, and CPB anticoagulation approaches

Comparators: Standard perioperative care or alternative regimens when reported

Outcomes: Perioperative bleeding, transfusion requirements, thrombotic complications, reoperation for bleeding, and perioperative mortality (in-hospital or ≤30 days)

Study designs: Randomized trials, observational cohorts, case series, and case reports

Exclusions: Pediatric-only studies; non-ITP thrombocytopenia; non-cardiac surgery; animal/in vitro studies; conference abstracts without full text; narrative reviews/editorials without primary patient-level data

No otherwise eligible full-text studies were excluded solely due to language or journal access restrictions.

Both primary and secondary ITP were eligible when ITP was explicitly diagnosed and considered the principal driver of perioperative thrombocytopenia. Mixed adult-pediatric reports were included only when adult (≥18 years) data were extractable; otherwise, they were excluded. Cardiac surgery was defined as open or minimally invasive operative cardiac procedures performed with or without CPB (including off-pump surgery); purely percutaneous or catheter-based interventions were excluded.

Information Sources and Search Strategy

No language restrictions were applied. Gray literature searching was limited to ClinicalTrials.gov and the WHO ICTRP; other gray sources (e.g., conference abstracts, theses, or preprints) were not systematically searched to minimize incomplete data capture and duplicate reporting in a predominantly case-based literature. The August 26, 2025, cutoff corresponds to the most recent literature available at the time of final manuscript revision and submission.

We systematically searched PubMed/MEDLINE, Embase, Scopus, Web of Science, and Cochrane CENTRAL from inception to August 26, 2025. Clinical trial registries, including ClinicalTrials.gov and the World Health Organization International Clinical Trials Registry Platform (WHO ICTRP), were also queried. The search strategy combined controlled vocabulary and free-text terms related to immune thrombocytopenia, cardiac surgery, CPB, and perioperative management.

Full database-specific search strategies are available upon request.

A representative search strategy was as follows:

("Immune Thrombocytopenic Purpura" OR "immune thrombocytopenia" OR ITP OR "idiopathic thrombocytopenic purpura" OR "autoimmune thrombocytopenia") AND ("cardiac surgery" OR "cardiothoracic surgery" OR "coronary artery bypass" OR CABG OR "valve surgery" OR "aortic surgery" OR "cardiopulmonary bypass" OR CPB) AND ("perioperative management" OR anticoagulation OR heparin OR bivalirudin OR antifibrinolytic* OR "platelet transfusion" OR IVIG OR steroid* OR "thrombopoietin receptor agonist" OR eltrombopag OR romiplostim OR bleeding OR transfusion OR thrombosis OR mortality)

Reference lists of included studies and relevant guideline documents were manually screened to identify additional eligible reports.

Screening was performed independently by two reviewers; however, blinding to authorship, institutions, or journals was not undertaken due to limited feasibility in a literature base dominated by case reports and small series. To mitigate selection bias, predefined eligibility criteria were applied uniformly, and disagreements were resolved by consensus or third-reviewer adjudication.

Study Selection

The timing of perioperative interventions relative to surgery (preoperative, intraoperative, or postoperative) was recorded when explicitly reported; however, precise temporal sequencing was inconsistently described and could not be systematically analyzed. Potential duplicate patient reports were assessed by cross-referencing author groups, institutions, study periods, procedural details, and patient characteristics; when overlap was suspected, only the most comprehensive or most recent report was retained.

Two reviewers independently screened titles/abstracts and then full texts. Disagreements were resolved by consensus or third-reviewer adjudication. Reasons for full-text exclusion were recorded.

Study screening was conducted independently by two reviewers; however, blinding to authorship, institutions, or journals was not performed, given the limited feasibility of blinding in a literature base dominated by case reports and small case series.

Formal inter-rater agreement statistics (e.g., kappa coefficients) were not calculated; discrepancies were resolved through discussion and, when necessary, adjudication by a third reviewer. Quality appraisal was used to contextualize interpretation rather than to exclude studies.

Data Extraction

Narrative synthesis followed the principles of the Synthesis Without Meta-analysis (SWiM) framework. Outcome proportions were calculated at the patient level using aggregated numerators and denominators derived from reported cases, without study-size weighting, formal hypothesis testing, or imputation of missing data; missing outcomes were handled by pairwise exclusion.

Because definitions of bleeding, transfusion thresholds, and the intensity of thrombosis surveillance varied substantially among reports, these figures should be interpreted as descriptive patterns rather than causal estimates.

Two reviewers independently extracted study characteristics, patient factors (including urgency and baseline platelet count), procedure type, perioperative interventions (steroids, IVIG, platelet transfusion, TPO-RAs, antifibrinolytics, anticoagulation/monitoring, point-of-care testing), and outcomes (bleeding, transfusion, thrombosis, reoperation, mortality) using a standardized form.

Risk-of-Bias Assessment

Given the heterogeneity of study designs, methodological quality was assessed using design-specific tools. Randomized trials, when applicable, were evaluated using the Cochrane Risk of Bias 2 (RoB-2) tool. Observational cohort studies were assessed using the Newcastle-Ottawa Scale. Case reports and case series were appraised using the CARE (CAse REport) checklist. Quality assessments were performed independently by two reviewers, with disagreements resolved by consensus.

Quality appraisal results were used to inform qualitative interpretation of findings and to contextualize confidence in reported management strategies and outcomes, rather than to determine study inclusion or exclusion, given the predominance of case reports and small case series. Formal inter-rater agreement statistics (e.g., kappa coefficients) were not calculated; disagreements in bias assessment were resolved through discussion and consensus, with adjudication by a third reviewer when necessary.

Synthesis Methods

Given heterogeneity across populations, procedures, and intervention bundles, we performed a narrative synthesis. Random-effects meta-analysis was prespecified for sufficiently comparable data; however, inconsistent outcome definitions and limited sample sizes constrained quantitative pooling.

Outcome proportions were calculated at the patient level using aggregated numerators and denominators derived from reported cases, without study-level weighting, formal hypothesis testing, or imputation of missing data.

Inferential statistics (e.g., P values or confidence intervals) were not reported, as their use would imply comparative inference not supported by the underlying evidence structure.

Narrative synthesis followed the principles of the Synthesis Without Meta-analysis (SWiM) framework, emphasizing transparent grouping of outcomes and structured presentation of results.

Results

Study Selection

**Figure 1:**
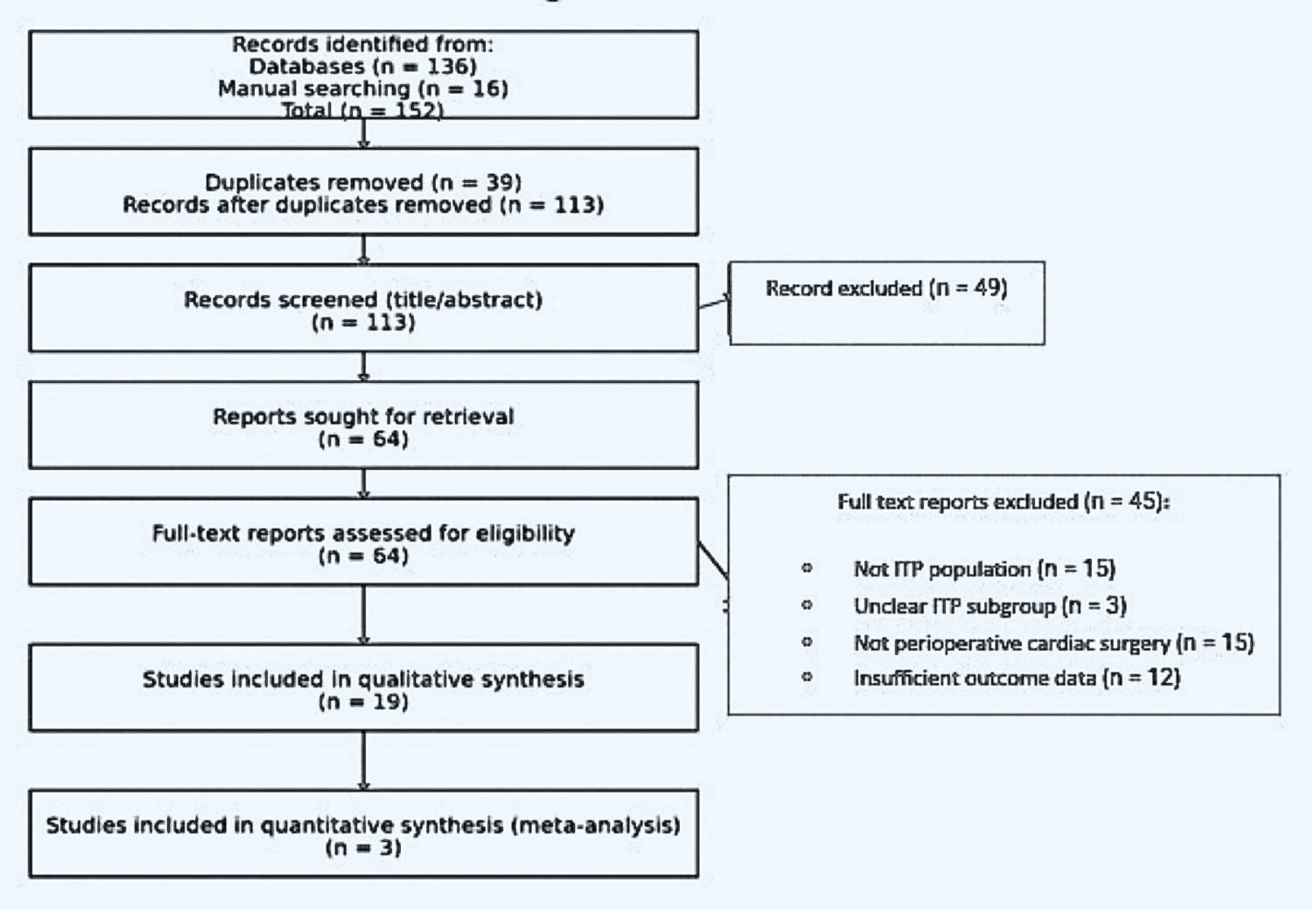
Preferred Reporting Items for Systematic Reviews and Meta-Analyses (PRISMA) 2020 flow diagram of the study selection A total of 152 identified; 39 duplicates removed; 113 screened; 49 excluded; 64 full texts assessed; 45 excluded; 19 included.152 identified; 39 duplicates removed; 113 screened; 49 excluded; 64 full texts assessed; 45 excluded; 19 included.

The systematic search yielded 152 records, including 136 identified through electronic databases and 16 through manual reference screening. Following the removal of 39 duplicates, 113 records underwent title and abstract screening, resulting in the exclusion of 49 records. Sixty-four full-text articles were subsequently assessed for eligibility, of which 45 were excluded based on prespecified criteria. Nineteen studies were ultimately included in the qualitative synthesis, comprising three observational cohort studies and sixteen case reports or small case series. The study selection process is summarized in Figure 1, which presents the PRISMA 2020 flow diagram.

Given the heterogeneity of study designs, methodological quality was appraised using design-appropriate tools: randomized trials, when present, were assessed with the Cochrane Risk of Bias 2 (RoB-2) tool; observational cohort studies with the Newcastle-Ottawa Scale; and case reports or case series with the CARE (CAse REport) checklist. All quality assessments were performed independently by two reviewers, with discrepancies resolved by consensus.

Where demographic data were reported, patients ranged from early adulthood to the eighth decade of life, with an approximately balanced sex distribution; however, inconsistent reporting limited meaningful aggregation. Baseline platelet counts and perioperative optimization strategies varied according to surgical urgency. Urgent or emergent cases more frequently involved severe thrombocytopenia, often below 30 × 10⁹/L, with minimal opportunity for staged preoperative augmentation, whereas elective cases more commonly described planned optimization to platelet counts in the 30-50 × 10⁹/L range. Across studies, platelet thresholds guiding immunomodulatory therapy or transfusion, perioperative platelet nadirs, and longitudinal platelet trajectories were inconsistently documented and appeared highly institution- or clinician-dependent. Included reports originated from multiple geographic regions and spanned the late 1990s through 2025, reflecting temporal evolution in surgical techniques, transfusion practices, and anticoagulation strategies.

Study Characteristics

The included studies comprised approximately 74 adults with ITP undergoing cardiac surgery, the majority presenting with severe thrombocytopenia (platelet count <50 × 10⁹/L). Reported procedures encompassed coronary artery bypass grafting, valve surgery, aortic root and ascending aortic operations, as well as combined and complex interventions, performed across elective, urgent, and emergent settings. Given the substantial heterogeneity in study designs, patient characteristics, and clinical contexts, the principal features of the included populations and interventions are systematically summarized in Table 1, providing a structured framework for interpretation of subsequent management strategies and outcome patterns.

**Table 1:** Study characteristics of the included studies

Characteristic	Summary
Included studies	19 (3 observational cohorts; 16 case reports/series)
Approximate patients	~74 adults with ITP
Baseline platelet count	Most <50 × 10⁹/L
Procedures	CABG; valve surgery; aortic/root/ascending aorta; combined/complex operations
Setting	Elective, urgent, and emergent across multiple regions

Perioperative Management Strategies

Because the included literature consisted predominantly of single-patient reports, small case series, and a limited number of observational cohorts, all outcome rates are reported as simple aggregated patient-level proportions rather than study-size-weighted estimates. Definitions of perioperative bleeding were heterogeneous and included clinically evident surgical bleeding, excessive chest tube output, transfusion beyond institutional norms, and/or reoperation for bleeding, depending on individual report criteria; standardized bleeding severity scales (e.g., BARC or TIMI) were rarely reported and were not applied consistently. When reported, platelet transfusion intent (prophylactic vs reactive) and the timing of thrombotic events (intraoperative vs. early postoperative vs late) were variably described, and mortality attribution was most often multifactorial.

Preoperative optimization most frequently relied on intravenous immunoglobulin (IVIG) and/or short-course corticosteroids to achieve rapid platelet augmentation, particularly in time-sensitive settings. Platelet transfusions were administered variably, often immediately prior to skin incision and/or after separation from CPB, according to bleeding severity and institution-specific thresholds. By contrast, thrombopoietin receptor agonists (eltrombopag or romiplostim) were primarily reported in elective contexts, where sufficient lead time allowed for staged platelet optimization. Intraoperatively, antifibrinolytic therapy (most commonly tranexamic acid) and structured PBM measures were frequently implemented. CPB anticoagulation was overwhelmingly conducted using activated clotting time-guided unfractionated heparin, with bivalirudin described only in isolated cases. Given the bundled and phase-specific nature of these interventions, the principal perioperative strategies are systematically organized by domain and timing in Table 2 and Table 3 to facilitate reproducible interpretation and clinical implementation.

**Table 2:** Perioperative management strategies reported in the included studies.

Phase/Domain	Strategies reported
Preoperative (urgent/emergent)	IVIG; short-course corticosteroids; selective platelet transfusion
Preoperative (elective)	TPO-RAs (eltrombopag/romiplostim) for staged platelet augmentation
Intraoperative adjuncts	Tranexamic acid; meticulous hemostasis; PBM measures; point-of-care testing when available
CPB anticoagulation	ACT-guided unfractionated heparin (dominant); bivalirudin (isolated)

**Table 3:** Perioperative management strategies Abbreviations: ACT, activated clotting time; CPB, cardiopulmonary bypass; IVIG, intravenous immunoglobulin; ROTEM, rotational thromboelastometry; TPO-RA, thrombopoietin receptor agonist

Author	Steroids	IVIG	Platelet transfusion	TPO-RA	Antifibrinolytic	CPB anticoagulation	Other adjuncts
Mathew et al. [8]	Yes	Yes	Yes	No	No	Heparin (ACT-guided)	—
Chowdhry et al. [9]	Yes	Yes	Yes	No	Yes	Heparin (ACT-guided)	—
Mallick et al. [10]	Yes	Yes	Yes	No	Yes	Heparin (ACT-guided)	—
Yasin et al. [11]	No	Yes	Yes	No	Yes	Heparin (ACT-guided)	—
Rossi et al. [12]	No	No	No	No	Yes	Heparin (ACT-guided)	ROTEM-guided management

Clinical Outcomes

Across the included studies, bleeding complications were reported in approximately 32% of patients, platelet transfusion in 45%, reoperation for bleeding in 8%, thrombotic events in 6%, and perioperative mortality in 7%, with consistently less favorable outcomes observed in urgent and emergent settings. Accordingly, these proportions should be interpreted as descriptive signals reflecting reported experience rather than comparative or causal estimates. Because definitions of bleeding, transfusion thresholds, and the intensity of thrombosis surveillance varied substantially among reports, these figures should be interpreted as descriptive patterns rather than causal estimates. Accordingly, outcome distributions are presented as aggregated proportions and urgency-stratified signals in Table 4 to facilitate contextualized interpretation.

**Table 4:** Outcomes summary and comparison by urgency

Outcome	Summary
Bleeding complications	~32% overall; higher signal in urgent/emergent
Platelet transfusion	~45% overall; more frequent urgent/emergent
Reoperation for bleeding	~8% overall; higher signal in urgent/emergent
Thrombotic events	~6% overall; vigilance required in both settings
Perioperative mortality	~7% overall; higher signal in urgent/emergent

Discussion

This systematic review synthesizes the available evidence on perioperative management strategies for adults with ITP undergoing cardiac surgery and underscores the intrinsic constraints of a literature base dominated by single-patient reports, small case series, and a limited number of observational cohorts. Although such designs offer valuable mechanistic insights and pragmatic guidance in rare and high-risk clinical scenarios, they inherently limit causal inference and preclude robust comparative effectiveness assessment.

To ensure strict adherence to PRISMA reporting standards and to avoid conceptual overlap, the Results section was limited to descriptive reporting of study characteristics, management strategies, and outcome proportions, whereas all mechanistic interpretation, contextualization, and clinical inference are consolidated within the Discussion section.

Nevertheless, despite marked heterogeneity in clinical contexts, baseline platelet counts, and therapeutic bundles, several convergent patterns emerge that support a coherent, multimodal perioperative paradigm centered on rapid immunomodulation when indicated, selective platelet support, routine antifibrinolytic therapy, structured PBM, and standardized anticoagulation monitoring during CPB. Importantly, these recurring patterns should be interpreted as descriptive signals rather than definitive therapeutic hierarchies, reflecting the predominantly non-comparative and selectively reported nature of the existing literature.

From a mechanistic perspective, ITP confers a bidirectional hemostatic vulnerability: immune-mediated platelet destruction and impaired production predispose patients to bleeding, whereas immune activation, endothelial perturbation, and inflammatory signaling may paradoxically promote thrombosis [1-3]. These competing risks are further amplified in the context of cardiac surgery, where CPB independently induces platelet activation, consumption, and qualitative dysfunction. Prothrombotic mechanisms described in ITP include platelet microparticle generation, immune complex-mediated endothelial activation, complement activation, and cytokine-driven hypercoagulability, which may persist despite thrombocytopenia and can be further potentiated by perioperative inflammatory stress and platelet-augmenting therapies. Notably, postoperative thrombocytopenia in this setting has been independently associated with increased morbidity and mortality [4]. However, the extent to which these perturbations interact synergistically or antagonistically in individual patients remains poorly characterized, owing to the absence of standardized platelet thresholds, inconsistent definitions of clinically relevant bleeding, and heterogeneous reporting of thrombotic surveillance.

Importantly, thrombotic event detection in the included literature was largely passive and clinically driven, with limited use of systematic screening, standardized timing, or uniform definitions, raising the possibility of underascertainment, particularly for subclinical or delayed thrombotic complications.

Accordingly, perioperative planning in ITP should be grounded in a balanced hemorrhagic-thrombotic risk framework rather than a bleeding-centric paradigm, while recognizing that precise individual risk stratification is currently constrained by these evidentiary gaps.

No standardized bleeding classification system (e.g., BARC or E-CABG criteria) was uniformly applied, limiting direct comparability of reported bleeding rates across studies.

Across the included literature, guideline-consistent first-line strategies-namely intravenous immunoglobulin (IVIG) and/or short-course corticosteroids-were most frequently employed to achieve rapid platelet augmentation, particularly in urgent and emergent scenarios where optimization time is limited [1,2]. In contrast, thrombopoietin receptor agonists (TPO-RAs) were predominantly used in elective contexts, reflecting their delayed onset of action and suitability for staged platelet optimization. This practice is supported by randomized evidence demonstrating noninferiority of eltrombopag compared with IVIG for achieving perioperative platelet targets [13]. However, cardiac surgical patients remain underrepresented in randomized ITP trials, and extrapolation from non-surgical populations must therefore be interpreted with caution. Platelet transfusion alone was rarely durable, consistent with immune-mediated clearance, but appeared to confer greater clinical utility when administered adjunctively following immunomodulation rather than as a standalone intervention [1-3]-a finding that, while biologically plausible, remains largely supported by uncontrolled observations.

The intraoperative period introduces additional complexity. Unfractionated heparin with activated clotting time-guided monitoring remains the dominant anticoagulation strategy for CPB, in accordance with contemporary practice guidelines [14]. Alternative agents such as bivalirudin were described only in isolated reports and remain constrained by monitoring complexity, lack of reversibility, and cost considerations [15,16]. Antifibrinolytic therapy-most commonly tranexamic acid-was frequently incorporated and is supported by high-level evidence demonstrating reductions in bleeding and transfusion in cardiac surgery [17].

Collectively, these components align naturally with PBM frameworks, which provide an organizing structure to minimize allogeneic exposure while maintaining hemostatic control [5-7]. It should be noted that explicit implementation of formal PBM programs was rarely described in the included studies; references to PBM herein reflect retrospective alignment of reported perioperative practices with PBM principles rather than systematic, protocolized PBM application. Nonetheless, the extent to which PBM principles can be systematically tailored to the immunopathophysiology of ITP remains incompletely defined, as most recommendations are extrapolated from general cardiac surgery populations rather than ITP-specific cohorts.

It should be emphasized that much of the supporting evidence for antifibrinolytic therapy, PBM frameworks, and thrombopoietin receptor agonists is derived from general cardiac surgery populations or non-surgical ITP cohorts; therefore, their application in cardiac surgery patients with ITP should be interpreted as contextual and extrapolative rather than disease-specific.

A consistent signal toward worse outcomes was observed in urgent and emergent surgical settings, plausibly reflecting the combined effects of limited time for staged platelet optimization, greater physiologic instability, and increased procedural complexity. In such contexts, rapid-acting regimens-IVIG with or without corticosteroids, supplemented by selective platelet transfusion-represent the most feasible therapeutic options [1,2], but must be accompanied by vigilant postoperative surveillance for both hemorrhagic and thrombotic complications. By contrast, elective surgery permits anticipatory multidisciplinary planning and staged optimization strategies, including the use of TPO-RAs, which may stabilize perioperative platelet trajectories and reduce transfusion burden. Importantly, these apparent differences between urgent and elective contexts should be viewed as hypothesis-generating rather than definitive, given the absence of controlled comparative data and the high likelihood of confounding by indication.

Importantly, the observed associations between surgical urgency and adverse perioperative outcomes in this review should be interpreted strictly as descriptive patterns rather than evidence of a causal relationship. Patients undergoing urgent or emergent cardiac surgery inherently differ from elective candidates with respect to baseline disease severity, hemodynamic instability, inflammatory burden, procedural complexity, and the presence of concurrent complications, all of which independently influence bleeding risk, thrombotic propensity, transfusion requirements, and mortality. This confounding by indication and severity, together with non-random treatment allocation, heterogeneous perioperative strategies, and inconsistent outcome reporting, precludes attribution of outcome differences to urgency status itself and reinforces the hypothesis-generating nature of these observations.

Crucially, the observed associations between surgical urgency and adverse outcomes in this review should not be interpreted as evidence of a causal relationship. Patients undergoing urgent or emergent cardiac surgery inherently differ from elective candidates with respect to baseline disease severity, hemodynamic instability, inflammatory burden, procedural complexity, and the presence of concurrent complications, all of which independently influence bleeding, thrombosis, and mortality risk. This confounding by indication and severity, coupled with non-random treatment allocation and heterogeneous reporting, precludes attribution of outcome differences to urgency status itself and underscores the descriptive nature of these findings.

Given the predominance of non-comparative, case-based evidence, the proposed perioperative management algorithm should be regarded as hypothesis-generating and expert-opinion-informed rather than evidence-validated or prescriptive.

To translate these heterogeneous observations into a reproducible and clinically applicable framework, we propose an urgency- and platelet-stratified perioperative management pathway integrating immunomodulatory strategies, transfusion principles, antifibrinolytic therapy, PBM measures, and standardized CPB anticoagulation, while allowing individualized risk calibration. Within this framework, perioperative decision-making is conceptualized as a sequential and risk-adaptive process that first differentiates patients according to surgical urgency (elective versus urgent/emergent) and subsequently refines management based on baseline platelet burden (<30, 30-50, or >50 × 10⁹/L). In urgent or emergent contexts, the pathway prioritizes rapid immunomodulatory interventions-typically IVIG with or without short-course corticosteroids-supplemented by adjunctive platelet transfusion when clinically indicated. These measures are integrated with antifibrinolytic therapy, comprehensive PBM strategies, and standard CPB anticoagulation using ACT-guided unfractionated heparin, followed by intensified postoperative surveillance for both hemorrhagic and thrombotic complications. By contrast, elective pathways emphasize staged platelet optimization, most commonly using TPO-RAs when sufficient lead time is available, with escalation to IVIG and/or corticosteroids in cases of inadequate response or evolving temporal constraints.

Accordingly, this framework is intended to function as a structured decision aid to support multidisciplinary planning, not as a validated clinical protocol or standard of care.

**Figure 2:**
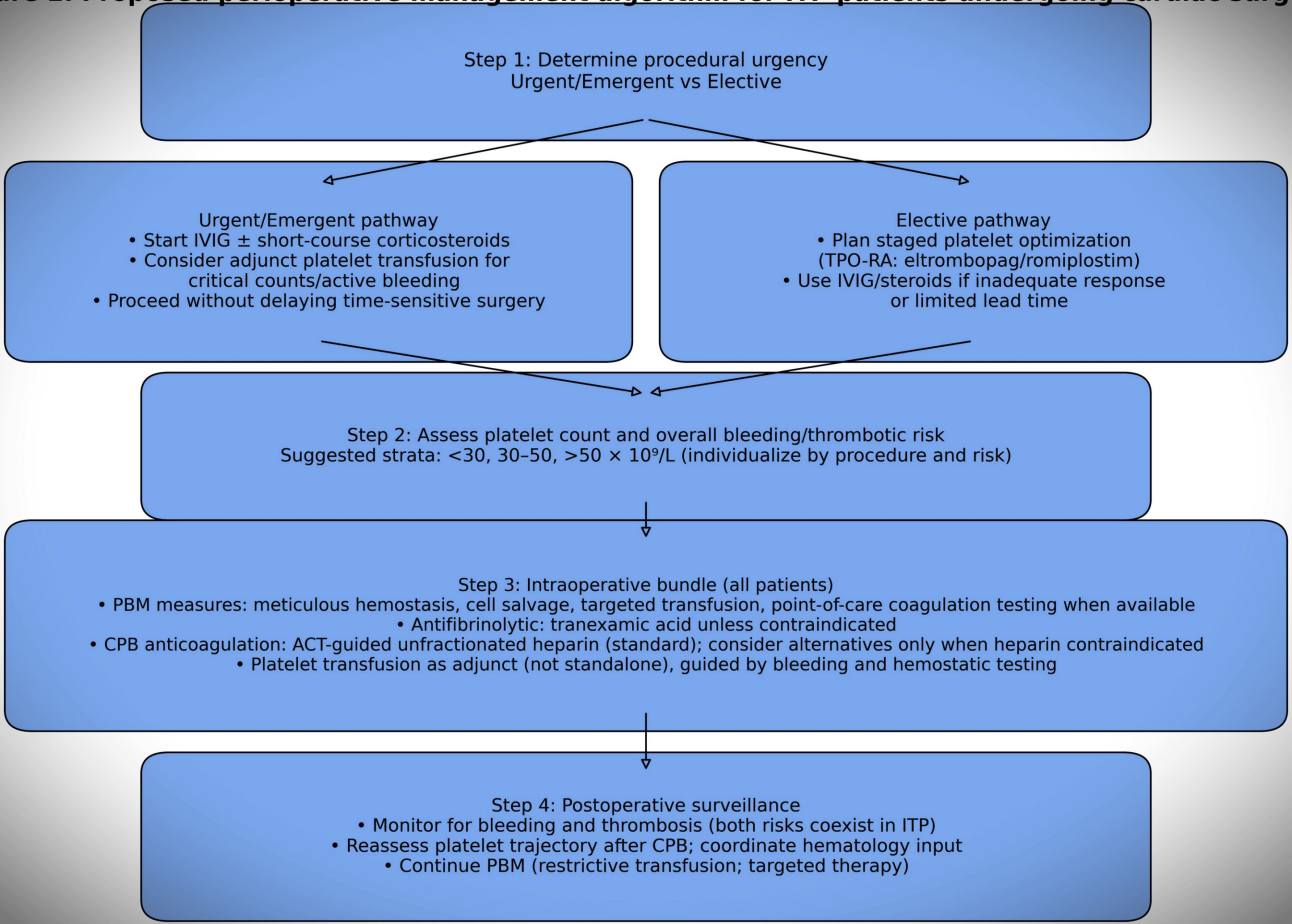
This algorithm is derived from a descriptive synthesis of published case-based experience and expert contextualization and has not been prospectively validated This algorithm stratifies patients according to surgical urgency, baseline platelet count, and individualized hemorrhagic–thrombotic risk. In urgent or emergent settings, management prioritizes rapid immunomodulation (intravenous immunoglobulin (IVIG) ± short-course corticosteroids), adjunctive platelet transfusion when indicated, antifibrinolytic therapy, patient blood management measures, and standard activated clotting time (ACT)-guided unfractionated heparin anticoagulation during cardiopulmonary bypass (CPB). In elective settings, the pathway emphasizes staged platelet optimization with thrombopoietin receptor agonists, with escalation to IVIG and/or corticosteroids when response is inadequate or timing becomes constrained. Image created by the authors with Microsoft PowerPoint (Microsoft Corp., USA) and Adobe Illustrator (Adobe, USA)

Framing perioperative care through this sequential, risk-adaptive lens enables harmonization of heterogeneous practices into a reproducible, patient-centered decision model (Figure 2).

By contrast, elective pathways emphasize staged platelet optimization, most commonly using thrombopoietin receptor agonists when sufficient lead time is available, with escalation to IVIG and/or corticosteroids in cases of inadequate response or evolving temporal constraints. This strategy is similarly embedded within a structured PBM framework, standardized intraoperative anticoagulation, and routine postoperative monitoring. Framing perioperative care through this sequential, risk-adaptive lens enables harmonization of heterogeneous practices into a reproducible, patient-centered decision model. In parallel, we summarize phase-specific interventions, their rationale, and the approximate level of supporting evidence in a practical bedside-oriented schema (Table 5).

**Table 5:** Practical perioperative considerations in patients with immune thrombocytopenic purpura undergoing cardiac surgery This table outlines phase-specific perioperative interventions for patients with immune thrombocytopenic purpura undergoing cardiac surgery, together with their clinical rationale and approximate level of supporting evidence, to support practical, multidisciplinary decision-making. Abbreviations: ACT, activated clotting time; CPB, cardiopulmonary bypass; IVIG, intravenous immunoglobulin; TPO-RAs, thrombopoietin receptor agonists

Phase	Intervention	Rationale	Level of evidence
Preoperative	IVIG ± corticosteroids	Rapid platelet augmentation	Low
Preoperative (elective)	TPO-RAs (eltrombopag/romiplostim)	Sustained platelet increase	Moderate
Intraoperative	Tranexamic acid	Reduction of fibrinolysis	High
Intraoperative	ACT-guided unfractionated heparin	Standard CPB anticoagulation	High
Postoperative	Bleeding and thrombosis surveillance	Early detection of complications	Low

Finally, it must be recognized that the current knowledge landscape is shaped by selective reporting, variable outcome definitions, non-uniform follow-up, and inconsistent thrombosis surveillance, all of which restrict causal inference and preclude robust quantitative synthesis in this review. Publication bias is likely, as atypical, complex, or particularly successful cases may be preferentially reported. Older studies further reflect historical transfusion practices, anticoagulation strategies, and surgical techniques that may not fully align with contemporary standards of care. Consequently, the patterns identified herein should be regarded as evidence-informed heuristics rather than definitive therapeutic mandates. Prospective, multicenter registries using harmonized outcome definitions and standardized perioperative reporting are urgently needed to refine platelet thresholds, optimize timing of interventions, and establish evidence-based perioperative pathways for this uniquely high-risk population.

Overall, risk-of-bias assessments indicated that most included evidence was of low to moderate methodological quality, reflecting the predominance of case reports and small series with inherent susceptibility to selection bias, incomplete outcome reporting, and confounding by indication. Although methodological quality was systematically appraised using design-appropriate tools, the interpretive impact of these assessments was necessarily qualitative, as most studies lacked a comparative design, standardized outcome definitions, or a sufficient sample size to permit stratification by risk of bias. Where observational cohort data of comparatively higher methodological rigor were available, these studies were preferentially emphasized in interpretation; however, no formal weighting or exclusion based on quality scores was applied, consistent with the descriptive and hypothesis-generating aims of this synthesis.

Although this review captures the relevant indexed literature available to date, the predominance of case reports and small series raises the possibility that unpublished, non-indexed, or institution-specific experiences were not captured, potentially underrepresenting real-world practice patterns and outcomes. Consequently, the patterns identified herein should be regarded as evidence-informed heuristics rather than definitive therapeutic mandates. Prospective, multicenter registries using harmonized outcome definitions and standardized perioperative reporting are urgently needed to refine platelet thresholds, optimize timing of interventions, and establish evidence-based perioperative pathways for this uniquely high-risk population.

The included studies span a broad temporal range (late 1990s-2025) and multiple geographic regions, which frames external validity and reflects progressive changes in perioperative anticoagulation, transfusion practice, and PBM. Despite cross-checking for overlap, unrecognized duplicate reporting cannot be fully excluded in older case-based literature with limited patient-level detail.

## Conclusions

The perioperative management of ITP in cardiac surgery represents a complex interplay between hemorrhagic and thrombotic risk, amplified by CPB and mandatory systemic anticoagulation. Despite reliance on predominantly case-based evidence, this systematic synthesis identifies consistent practice patterns supporting a multimodal strategy integrating rapid immunomodulation when time is limited, selective platelet transfusion, antifibrinolytic therapy, structured PBM, and carefully monitored anticoagulation. Outcomes appear consistently worse in urgent and emergent settings, underscoring the importance of anticipatory planning, multidisciplinary coordination, and intensified postoperative surveillance, while elective procedures allow more deliberate optimization. Although current evidence precludes definitive treatment hierarchies, emerging immunomodulatory therapies and standardized blood management frameworks offer an opportunity to transform perioperative ITP care from an ad hoc challenge into a protocol-driven, outcome-oriented practice, supported by prospective multicenter registries using harmonized outcome definitions.
